# Stemming the Rising Tide of Human-Biting Ticks and Tickborne Diseases, United States

**DOI:** 10.3201/eid2610.201271

**Published:** 2020-10

**Authors:** Andrea Egizi, Robert A. Jordan

**Affiliations:** Tick-borne Disease Program, Monmouth County Mosquito Control Division, Tinton Falls, New Jersey, USA

**Keywords:** tick-borne diseases, mosquito-borne diseases, ticks, mosquito control, mosquitoes, local government, vector-borne infections, United States

**To the Editor:** We agree with Eisen (*1*) that local/county vector control agencies (VCAs) are well-positioned to address tickborne disease prevention. However, addressing tickborne diseases using VCAs requires substantial long-term support from local administrators and taxpayers and would necessitate changing the way vector control programs are currently funded to a more proactive approach.

Sustainable funding is critical because ticks rebound quickly when management efforts cease (*2*). Many VCA budgets are eroded in the years between mosquitoborne disease outbreaks, leaving them ill-prepared for the next outbreak (*3*). Consequently, tickborne disease programs could experience major setbacks if their resources are redirected during a mosquitoborne disease outbreak.

Eisen acknowledges (*1*) that known barriers to implementation of community-based tick control include a lack of optimized best practices for tick suppression that link reductions in tick populations to measurable reductions in human disease, as well as the lack of real-world cost estimates for their implementation. Tickborne disease programs without proper budgets and realistic expectations that purport to reduce incidence but fail to do so (or fail to do so quickly) run the risk of undermining public trust and willingness to sustain funding.

Last, we caution that managing ticks in residential situations (as opposed to high-risk public open spaces and trails) is fraught with technical and public relations challenges, legal issues, and likely insurmountable funding demands (*4*,*5*). The complex array of environmental and social factors contributing to the increase in tickborne disease cases (e.g., forest management practices, climate change, land use, and an aging population) is frankly beyond the scope of any individual VCA to address without higher-level (state and federal) coordination.

A proactive approach with higher-level coordination will help manage tickborne disease. To give VCAs the best chance to combat tickborne disease, they must be adequately and sustainably funded to manage mosquitoes and ticks, even during years of fiscal challenge.
